# FcγRIIIA Genotype in Plasma Cell Dyscrasias Is Associated with Clinical Progression, Bone Disease Extension and Immune Dysfunction

**DOI:** 10.3390/cancers18071084

**Published:** 2026-03-26

**Authors:** Daniela Cambria, Maria Teresa Cannizzaro, Nunziatina Laura Parrinello, Sara Marino, Ilaria Dulcamare, Noemi Puccio, Federica Torricelli, Marta Lionetti, Deborah Calvo, Mohammadreza Khosropoor, Concetta Conticello, Francesco Di Raimondo, Lavinia Raimondi, Gianluca Giavaresi, Cirino Botta, Antonino Neri, Alessandra Romano

**Affiliations:** 1 Department of Health Promotion, Mother and Child Care, Internal Medicine and Medical Specialties, University of Palermo, 90133 Palermo, Italy; daniela.cambria@policlinico.unict.it (D.C.);; 2 UOSD Radiologia, AOU Policlinico Rodolico San Marco, 95123 Catania, Italy; 3 UOC Ematologia, AOU Policlinico Rodolico San Marco, 95123 Catania, Italy; 4 Dipartimento di Chirurgia e Specialità Medico Chirurgiche, Università Degli Studi di Catania, 95123 Catania, Italy; 5 Laboratory of Translational Research, Azienda USL-IRCCS di Reggio Emilia, 42123 Reggio Emilia, Italy; 6 Department of Oncology and Hematology-Oncology, University of Milan, 20122 Milan, Italy; 7 Scienze e Tecnologie Chirurgiche, IRCCS Istituto Ortopedico Rizzoli, 40136 Bologna, Italy; lavinia.raimondi@ior.it (L.R.); gianluca.giavaresi@ior.it (G.G.); 8 Scientific Directorate, Azienda USL-IRCCS di Reggio Emilia, 42123 Reggio Emilia, Italy

**Keywords:** MGUS, SMM, multiple myeloma, inflammatory monocytes, bone disease, FcγRIIIA, polymorphism

## Abstract

**Background/Objectives**: FcγRIIIA presents a single nucleotide polymorphism at position 158 (V/F), which affects its binding affinity to the fragment crystallizable (Fc) of antibodies (Abs). In the presence of immune complexes, FcγRIIIA can mediate the inflammatory signaling, severity of bone disease, and osteoclastogenic activity. Based on this functional relevance, we hypothesized that the FcγRIIIA F158V polymorphism may influence the clinical presentation of multiple myeloma (MM). **Methods**: FcγRIIIA F158V genotyping was performed on genomic DNA extracted from peripheral blood samples of patients affected by MM or asymptomatic conditions named MGUS and SMM. We compared the allele frequency of FcγRIIIA-F158V polymorphism in 72 MM, 42 MGUS and 31 SMM and evaluated the association with clinical features and occurrence of high-risk chromosome abnormalities. Targeted NGS mutation analysis was performed on genomic DNA isolated from purified CD138^+^ bone marrow plasma cells (BMPCs) of 41 patients, to evaluate the association between somatic mutations and the FcγRIIIA F158V genotype. **Results**: the FcγRIIIA-158 V/V homozygous genotype was associated with high-risk cytogenetics, anemia, high beta-2 microglobulin levels, and more than 10 osteolytic lesions. V/V homozygous genotype was significantly associated with at least one mutation in RAS pathway genes (*N-RAS*, *K-RAS* or *B-RAF*). In the immune microenvironment, patients carrying the V/V homozygous genotype had a higher percentage of CD14^+^CD16^++^ non-conventional inflammatory monocytes than the V/F or FF genotype. **Conclusions**: Our study contributes to a better understanding of the interactions between genetic variants, tumor microenvironment, and therapeutic response in plasma cell dyscrasias, to identify molecular biomarkers for precision medicine in MM, MGUS and SMM.

## Simple Summary

The survival and growth of myeloma cells depend not only on their own genetic mutations but also on the patient’s immune system. Host immunogenetic variability represents a potential but underexplored determinant of disease evolution and risk stratification in plasma cell disorders. In this study, we investigated how a specific genetic variation in an immune receptor (FcγRIIIA-158 V/V) affects the clinical course of plasma cell diseases, from early stages (MGUS) to overt multiple myeloma. We found that patients with the V/V variant have a much higher risk of developing aggressive disease features, including extensive bone damage and high-risk genetic mutations (*NRAS*, *KRAS*, or *BRAF* in the MAPK pathway) and exhibited an expanded population of pro-inflammatory monocytes, which can contribute to chronic inflammation and immune senescence onset. These findings are important because they identify a new, low-cost genetic marker that can help doctors predict which patients are at higher risk of disease progression, potentially allowing for more personalized monitoring and treatment.

## 1. Introduction

Multiple myeloma (MM) is a hematological cancer due to the aberrant expansion of neoplastic plasma cells (PC) within the bone marrow (BM). The concomitant presence of at least 10% of PC in BM and any organ damage (hypercalcemia, renal failure, anemia, or osteolytic lesions of the bone) identifies patients with symptomatic MM who require frontline treatment [1]. Pre-malignant phases of MM include asymptomatic conditions characterized by a limited infiltration of PCs in the BM as monoclonal gammopathy of uncertain significance (MGUS) and smoldering myeloma (SMM) [2,3,4].

One of the hallmark features of MM is the associated bone disease, manifested as lytic lesions, osteopenia, and fractures, detected through imaging studies such as X-rays, computed tomography (CT) scans, or magnetic resonance imaging (MRI), occurring in over 80% of patients with newly diagnosed MM, responsible for significant morbidity and mortality [5].

The onset of MM-related bone disease is a complex process involving dysregulation of bone remodeling, characterized by increased osteoclastic bone resorption and suppressed osteoblastic bone formation [6]. The interaction between immunoglobulins, immune cells, and osteoclasts is highly complex and still not fully understood in myeloma bone disease.

In mice and humans, immune complexes (ICs) or aggregates of immunoglobulins (IgGs) with an altered pattern of glycosylation can promote osteoclast differentiation [7]. ICs can be recognized and cleared by the immune system through specific receptors able to bind to the constant fraction region of immunoglobulins [8] (FcγRs). FcγRs are membrane proteins expressed on various cells of the immune system and play a crucial role in regulating antibody-mediated responses [9]. Their primary function is to mediate binding to IgG immunoglobulins, modulating processes such as phagocytosis, antibody-dependent cellular cytotoxicity (ADCC), and cytokine release. In humans, FcγRs are classified into subclasses: FcγR1 (CD64), FcγR2 (CD32), and FcγR3 (CD16), each of which can exist as polymorphic variants due to mutations in their extracellular domains. For FcγR2A, the substitution of histidine (H) with arginine (R) at position 131 modifies the ability to bind specific IgG subclasses; whereas for FcγR3A the substitution of valine (V) with phenylalanine (F) at position 158 determines variable affinity for IgG1 and IgG3, directly influencing the effector functions of NK cells and macrophages [10,11]. Targeting FcγRIII with a specific antibody or natural IgG produces an inhibitory signal through FcγRIII in both mice and humans, suggesting that free IgG and IVIg help suppress inflammation [12,13,14].

While in the setting of inflammatory non-neoplastic disease like rheumatoid arthritis, IgG immune complexes (ICs) can activate FcγRs (especially FcγR3) on osteoclast precursors and enhance osteoclast differentiation via ITAM signaling [15,16], in MM ICs can modulate bone demineralization [17], or may promote lesion progression, through the binding of C-reactive protein to FcγR2A on myeloma cells [18].

However, little is known about the clinical relevance of pathological immunoglobulin in mediating bone disease in MM. To fill this gap in knowledge, we evaluated FCγR3A polymorphisms in a cohort of MGUS, smoldering myeloma, and active myeloma subjects, assessing their potential impact on disease predisposition and clinical presentation.

## 2. Methods

### 2.1. Patient Selection

Between October 2023 and May 2025, 72 consecutive newly diagnosed MM, 31 SMM and 42 MGUS patients were included in this study. This study was approved by the local Ethics Committee; protocol code: 60/2023/PO, study codename: PNRR-2022 12376660. Informed consent was obtained from all subjects involved in this study. Patients’ characteristics are summarized in Table 1. None of the recruited patients were receiving medical treatments that could have an impact on their immune condition. All subjects were devoid of immune-mediated diseases and acute or chronic viral infections to avoid any interference with immune-regulatory mechanisms.

Bone marrow aspiration and biopsy were performed at diagnosis in all patients based on clinical suspicion of progression or according to the IMWG risk stratification (i.e., serum M-protein ≥ 1.5 g/dL or non-IgG isotype). FISH for risk assessment included at least the research of 17p13 deletion, 1q gain, t (4;14), according to institutional protocols at the time of diagnosis, and was evaluable in 26 MGUS, 20 SMM and 60 MM patients, as detailed in Table 1. Next-Generation Sequencing (NGS) was performed only in 42 patients due to the limited availability of CD138^+^ cell sorting during the early recruitment period.

All subjects provided consent to use their medical records for research, in accordance with the Declaration of Helsinki.

A REDCap-based tracking system was implemented to support genetic evaluations, phenotyping, diagnostic testing, and follow-up care. The system collected demographic information, clinical features, and genetic test results. Data fields were designed with multiple-choice and calendar formats to standardize collection and reporting for commonly requested data.

### 2.2. Bone Lesion Detection via Whole-Body Low-Dose CT (WBLDCT)

Bone osteolytic lesions were assessed using whole-body low-dose CT in all subjects included in this study. All WBLDCT exams were performed on a 16-detector CT scanner (Philips Healthcare, Best, Netherlands) using the following parameters: tube voltage (kV)/time-current product (mAs) of 120/60; collimation 16 × 1.5 mm; pitch 0.94; rotation time 0.50 s. All patients were scanned from the cranial vertex to the proximal tibia with the arms positioned either above the head with as little bending at the elbows as possible, or on top of the abdomen with hands folded and the humeri included in the field of view. Source images were reconstructed with a high-frequency reconstruction algorithm for the detection of osteolytic lesions and a smooth, soft tissue reconstruction algorithm for evaluation of the medullary cavities of the appendicular skeleton and for soft tissue assessment.

### 2.3. Image Analysis

All CT exams were evaluated in consensus by two experienced radiologists (MTC and AB with 5 years of experience in interpreting WBLDCT scans of patients with plasma cell dyscrasias), who were blinded to the clinical and laboratory data. Image analysis was performed on a dedicated workstation using the Intellispace Portal diagnostic software ver.12 (Philips Healthcare, Best, Netherlands). The criteria for the identification of MM-related bone disease were the presence of at least one well-defined lytic lesion of the trabecular bone (≥5 mm) with no sclerotic margins, in line with established guidelines [4,19]. Hyperdense medullary lesions of the appendicular skeleton and fractures (including vertebral compression fractures) were also recorded but were not used for determining the presence of MM-related bone disease. Incidental findings, unrelated to myeloma, were also identified and recorded.

### 2.4. Phenotyping Monocytes by Flow Cytometry

Vein puncture blood in EDTA tubes was processed within two hours of sample collection.

For the evaluation of I.M., 1 × 10^6^ cells were stained with 10 µL of the following monoclonal antibodies (MoAbs) against: CD14, CD16, and CD45. After lysing red cells with ammonium chloride, cells were analyzed by flow cytometry, using an EPICS-XL (Beckman Coulter, Brea, CA, USA).

The following anti-human antibodies were purchased from Beckman Coulter: CD14 PE (clone RMO52), CD16 FITC (clone 3G8), and CD45 ECD (clone J.33). Isotype monoclonal antibodies were used as negative controls.

Monocytes were gated in a side light scatter SSC/CD14 dot plot, and inflammatory monocytes were distinguished by coexpression of CD14 with Fcγ-III receptor CD16a (CD14^+^/CD16^+^). We did not implement fluorescence-minus-one controls or bead-based calibration to standardize CD16 thresholds; therefore, modest misclassification of CD14^+^CD16^++^ events cannot be excluded.

### 2.5. DNA Isolation and Quantification

Each subject enrolled in this study provided 10 mL of peripheral blood from which white blood cells were isolated by hypotonic lysis.

Isolated white blood cells were stored as cell pellets for subsequent molecular analyses. Cell pellets from each patient were used to extract genomic DNA by salting-out technique.

The first step involves cell lysis, which allows the release of the DNA; following lysis, the next step is to remove proteins and other cellular debris that may interfere with DNA isolation. A protein precipitation reagent such as sodium dodecyl sulfate (SDS) and proteinase K was added to the lysate. These reagents break down protein structures and precipitate them out of solution. After the proteins were removed, a high concentration of salt, saturated sodium chloride (NaCl), was added to the solution. An equal volume of cold ethanol or isopropanol was then added to the mixture. The addition of alcohol further reduces the solubility of the DNA, causing it to clump together and form visible clumps. After allowing the DNA to precipitate, the mixture was centrifuged at high speed. Centrifugation forces the DNA to sink to the bottom of the tube, while the rest of the solution, including contaminants, remains in the supernatant. After centrifugation, the supernatant was carefully removed, and the DNA pellet was washed by adding a cold 70% ethanol solution to remove any residual contaminants. The DNA pellet was centrifuged again to remove ethanol, air-dried for a few minutes, and finally resuspended in Tris-EDTA buffer (TE).

The purity and concentration of the DNA obtained were determined through 260/280 nm absorbance measures using the NanoDrop^®^ ND-1000 UV-Vis Spectrophotometer (Thermo Scientific, Walthaman, MA, USA).

### 2.6. Real-Time PCR and Allelic Discrimination

We used the TaqMan^®^ SNP Genotyping Assays C__25815666_10 SNP ID: rs396991 for detecting FcγR3A V158F, following the manufacturer’s instructions.

The reaction mix consisted of TaqMan^®^ Genotyping Master Mix and TaqMan^®^ SNP Genotyping Assay according to protocol. The temperature profile of the reaction included 10 min at 95 ◦C and 40 amplification cycles (15 s 95 °C, 1 min 60 °C). Experiments were conducted using 7900HT Fast Real-Time PCR System (Applied Biosystems, Foster City, CA, USA) in 96-well plates. In total, 10 ng of genomic DNA were used in a total reaction volume of 25 µL.

Workflow involved three steps: a pre-read of the plate to record the background fluorescence of each well of the plate before absolute quantification PCR. The amplification was conducted according to the thermal profile described above, and finally post-PCR plate reading was performed. Allelic discrimination analysis was performed using SDS software (Applied Biosystems).

### 2.7. Next-Generation Sequencing

We collected tumor gDNA from CD138^+^-purified BM PCs. For panel sequencing, libraries were prepared using the Kapa HyperPrep kit (Roche, Madison, WI, USA) starting with 100 ng of gDNA. For ultra-deep mutation analysis 8 pre-capture libraries were pooled together in equal amounts, reaching a final quantity of 1500 ng. From each pre-capture pool, we performed target enrichment by probe hybridization using the HyperCap kit (Roche, Madison, WI, USA) for a custom panel of 56 genes. The targeted resequencing gene panel included coding exons and splice sites of 56 genes that emerged as drivers from a recent analysis of whole-genome and exome data in more than 800 MM patients [20] (target region (112 kb): *ACTG1*, *BCL7A*, *BHLHE41*, *BRAF*, *BTG1*, *CCND1*, *CDKN1B*, *CYLD*, *DIS3*, *DTX1*, *DUSP2*, *EGR1*, *FAM46C*, *FGFR3*, *FUBP1*, *HIST1H1B*, *HIST1H1D*, *HIST1H1E*, *HIST1H2BK*, *IGLL5*, *IRF1*, *IRF4*, *KLHL6*, *KMT2B*, *KRAS*, *LCE1D*, *LTB*, *MAX*, *NFKB2*, *NFKBIA*, *NRAS*, *PABPC1*, *PIM1*, *POT1*, *PRDM1*, *PRKD2*, *PTPN11*, *RASA2*, *RB1*, *RFTN1*, *RPL10*, *RPL5*, *RPRD1B*, *RPS3A*, *SAMHD1*, *SETD2*, *SP140*, *TBC1D29*, *TCL1A*, *TGDS*, *TP53*, *TRAF2*, *TRAF3*, *XBP1*, *ZNF462*, *ZNF292*).

Sequencing was performed on Miseq instrument (Illumina, San Diego, CA, USA), and 16 samples (2 pools) were sequenced together in a Miseq V3 cartridge (2 × 200 cycles).

Variant calling analysis was performed using the DRAGEN Somatic pipeline (Illumina BaseSpace Hub), and elaboration was performed using the Maftools R library. To focus on somatic and pathogenic variants, only variants passing Illumina filters were selected and further filtered for variant allele frequency < 5% and gnomAD frequency < 1%. Finally, synonymous and intronic variants were excluded.

### 2.8. Statistical Methods

Statistical analyses were performed using GraphPad Prism version 8.00 for Windows, GraphPad Software, San Diego, CA, USA. Continuous variables were expressed as medians and interquartile ranges (IQR), and comparisons between the three FcγRIIIA-158 genotypes (F/F, V/F, and V/V) were performed using the Kruskal–Wallis test. Categorical variables were summarized as counts and percentages, and associations were evaluated using the unpaired *t*-test with Welch’s correction, chi-square test or Fisher’s exact test, as appropriate. To account for the inflation of Type I error due to multiple hypothesis testing across 12 clinical and biological variables, a Bonferroni correction was applied, setting the threshold for formal statistical significance at *p* < 0.0041 (0.05/12). All *p*-values are two-sided, and those maintaining significance after correction are explicitly indicated in the text and tables.

## 3. Results

### 3.1. Frequency of FcγR3AV158F Genotype in MGUS, Smoldering, and Active Multiple Myeloma

**Figure 1:**
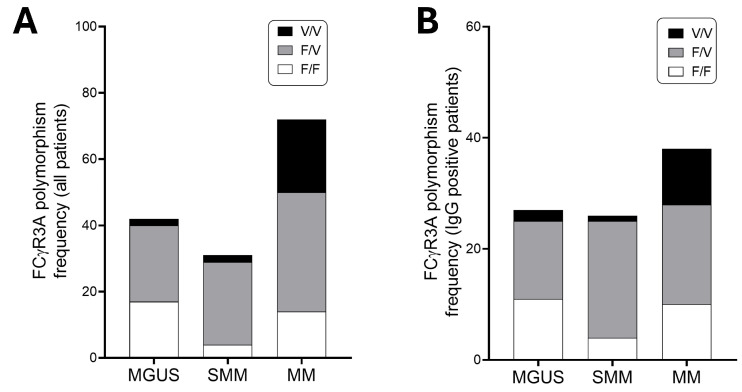
Frequency of FcγR3AV158F genotype in MGUS, smoldering and active multiple myeloma: (**A**) Frequency of FcγR3AV158F genotype in MGUS, smoldering and active multiple myeloma patients of the whole cohort. (**B**) Frequency of FcγR3AV158F genotype in MGUS, smoldering and active multiple myeloma patients carrying an IgG M-protein.

As shown in Figure 1A, among MGUS patients (*N* = 42), FcγR3AV158F polymorphism frequencies were 4.8% for FcγR3A-158 V/V homozygous genotype, 54.8% for FcγRRR3A-158 F/V heterozygous genotype and 40.5% for FcγR3A-158 F/F homozygous genotype. Among SMM patients (*N* = 31), FcγR3AV158F polymorphism frequencies were 6.5% for FcγR3A-158 V/V homozygous genotype, 80.0% for Fcγ3A-158 F/V heterozygous genotype and 12.9% for FcγR3A-158 F/F homozygous genotype. Among MM patients (*N* = 72), FcγR3AV158F polymorphism frequencies were 30.6% for FcγR3A-158 V/V homozygous genotype, 50.0% for Fcγ3A-158 F/V heterozygous genotype and 19.4% for FcγR3A-158 F/F homozygous genotype.

The distribution of FCGR3A-158V/F genotypes in our cohort (F/F: 24.1%; V/F: 57.9%; V/V: 17.9%) was found to be in Hardy–Weinberg Equilibrium (χ^2^ = 3.01, *p* = 0.083). When compared to the European population frequencies reported in the 1000 Genomes Project (F/F: 42%; V/F: 44%; V/V: 14% [21]), our cohort showed a higher prevalence of the V allele (46.9% vs. 36%). This enrichment of the high-affinity V variant in patients with plasma cell dyscrasias is consistent with previous reports suggesting that host immune genetics may influence disease susceptibility and clinical presentation [22].

Limiting the analysis to patients with IgG M-protein carriers (*N* = 91, including 27 MGUS, 26 SMM, and 38 MM), MM was enriched for V/V versus MGUS or SMM (χ^2^ 13.01, *p* = 0.001) within our cohort, acknowledging potential confounding by unmeasured ancestry, as shown in Figure 1B. We could not collect genetic ancestry or include matched healthy controls; therefore, case–case frequency differences may reflect unmeasured population structure.

### 3.2. FcγR3A-158 V/V Homozygous Genotype Is Associated with Adverse Clinical Features, Including Bone Disease Extension

**Figure 2:**
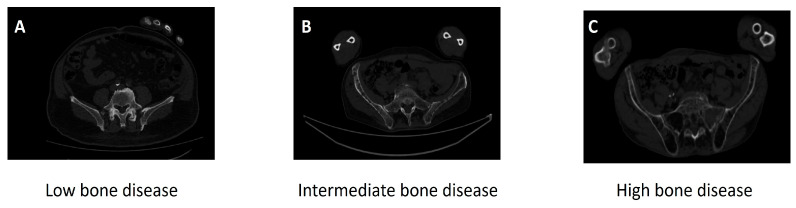
Bone disease detected by WBLDCT scans: In (**A**) axial reconstructions at the fifth lumbar body demonstrated along the pedicles and transverse processes widespread osteolytic involvement. Lesions are typically non-sclerotic and range in presentation from multiple discrete punched-out lucencies to more advanced, coalescent regions of bone destruction. (**B**) Axial WBLDCT image in bone window setting at the level of the sacral metamers and iliac wings. The bilateral iliac bones demonstrate extensive and diffuse involvement with numerous poorly defined, small, non-sclerotic osteolytic lesions, resulting in a general loss of bone density. Multiple focal punched-out lucencies are scattered throughout the trabecular bone, consistent with diffuse myeloma infiltration of the pelvic bone marrow. In (**C**) axial CT plane through the mid-pelvis reveals confluent areas of medullary destruction involving sacral metamers and bilateral sacroiliac joints. The trabecular pattern is largely replaced by a diffuse osteolytic process with significant endosteal scalloping particularly in the right sacral wing end bilateral iliac bone adjacent to the sacroiliac joint. A large destructive osseous lesion involving the left sacral wing “mini-brain” appearance, suggesting soft tissue plasmacytoma.

The distribution of FcγRIIIA-158 genotypes and their association with baseline characteristics are summarized in Table 2. Patients harboring the homozygous V/V genotype exhibited a significantly more aggressive clinical phenotype compared to V/F and F/F carriers. Specifically, the V/V genotype was strongly associated with advanced bone disease, with 84.6% of patients presenting 4 or more osteolytic lesions compared to only 14.3% in the F/F group (*p* < 0.0001). Figure 2 shows the gradient of osteolytic lesions in NDMM patients.

This clinical aggressiveness was further mirrored by a significant reduction in median hemoglobin levels (10.3 g/dL vs. 12.6 g/dL in F/F; *p* = 0.002) and a higher prevalence of high-risk cytogenetic abnormalities (53.8% vs. 5.7%; *p* < 0.0001).

Limiting analysis to the cohort of 72 newly diagnosed MM patients, the FcγR3A-158 V/V genotype was more frequent in patients carrying adverse prognostic factors at baseline, including lower hemoglobin, lower albumin, higher beta-2 microglobulin, higher LDH, more than 10 osteolytic lesions, high-risk FISH and extra medullary disease, as shown in Table 3.

Most notably, in the subset of 41 (12 MGUS, 9 SMM and 20 MM) patients with available NGS data, where we collected enough plasma cells for downstream analysis, the V/V genotype was highly predictive of MAPK pathway mutations. Two-thirds (66.6%) of V/V patients carried at least one mutation in NRAS, KRAS, or BRAF, compared to 22.7% and 20.0% in the V/F and F/F groups, respectively (*p* < 0.0001). Even after the stringent Bonferroni correction for multiple testing, the associations with bone disease, high-risk cytogenetics, and MAPK mutational status remained highly significant (*p_adj_* < 0.001), supporting the notion that mutations are present throughout the natural history of plasma cell dyscrasias (Table 4).

**Figure 3:**
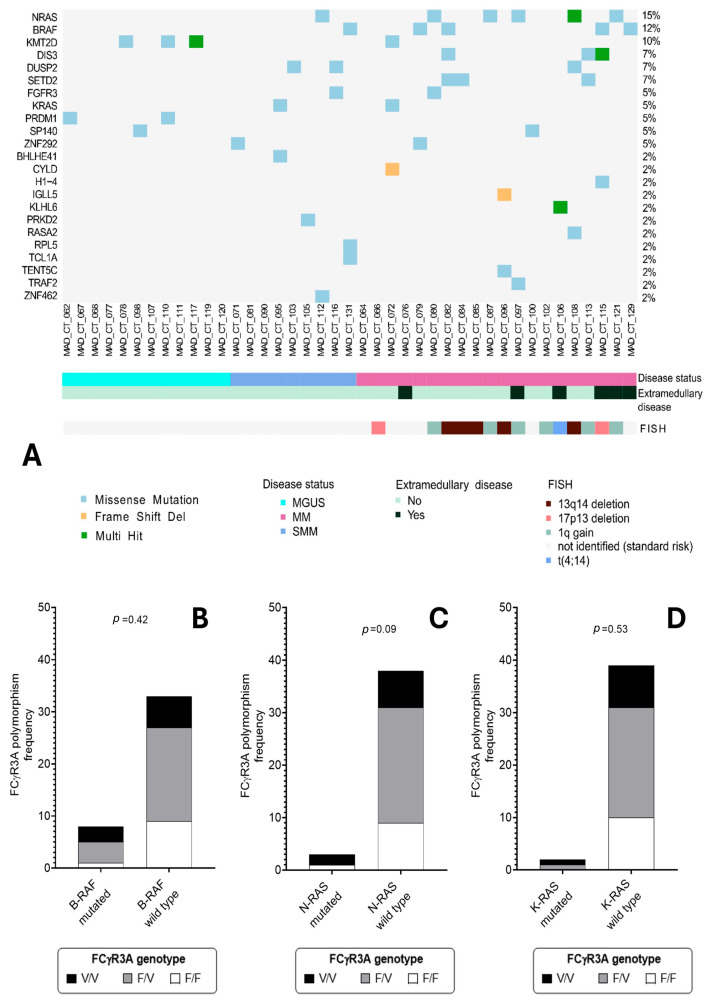
Mutational landscape of a selected cohort of patients and association with FcγRIIIA genotype. In (**A**) the waterfall plot shows for each patient the cytogenetic aberrations, detected by FISH, and specific somatic mutations detected by ultra-deep targeted NGS. The frequency of FcγR3AV158F genotype is reported separately for patients with mutated or wild-type B-RAF (**B**), N-RAS (**C**) and K-RAS (**D**), as detected by NGS. Groups are compared using the ANOVA test; differences in (**B**–**D**) are not statistically significant.

NRAS was the most frequently altered gene across the three stages. Despite not being a statistically significant difference, a higher frequency of NRAS mutations was detected in MM versus MGUS-SMM (25% vs. 4.8%). Similar frequencies were observed for BRAF alterations (20% in MM versus 4.8% in MGS-SMM). Notably, four out of six EMD-positive patients showed NRAS or BRAF mutation. The DIS3 gene was mutated in 15% of MM, while no mutations were observed in MGUS-SMM, and the same mutational frequency was found for the SETD2 gene (Figure 3A).

The V/V genotype was significantly associated with a higher frequency of mutations within the MAPK pathway (including NRAS, KRAS, or BRAF; *p* < 0.0001, Fisher’s exact test). However, this association was observed for the pathway as a whole, rather than being restricted to any single specific mutation (Figure 3B–D). This exploratory signal requires confirmation after adjustment for stage and tumor fraction, using matched germline and orthogonal assays.

The presence of at least one V allele was associated with increased relative risk of 2.4 (95% CI: 1.8 to 3.3), with 0.79 sensitivity (95% CI:0.71 to 0.86) and 0.94 specificity (95% CI:0.81 to 0.98) of carrying high-risk cytogenetics but not with a significant risk of having MGUS, SMM or MM disease (*p* = 0.88). Interestingly, the presence of at least one V allele was associated with increased relative risk of extensive bone disease of 2.5 (95% CI: 1.1 to 5.9), with 0.86 sensitivity (95% CI: 0.7 to 0.9).

### 3.3. CD14^+^CD16^++^ Inflammatory Monocytes Are Increased in Multiple Myeloma

**Figure 4:**
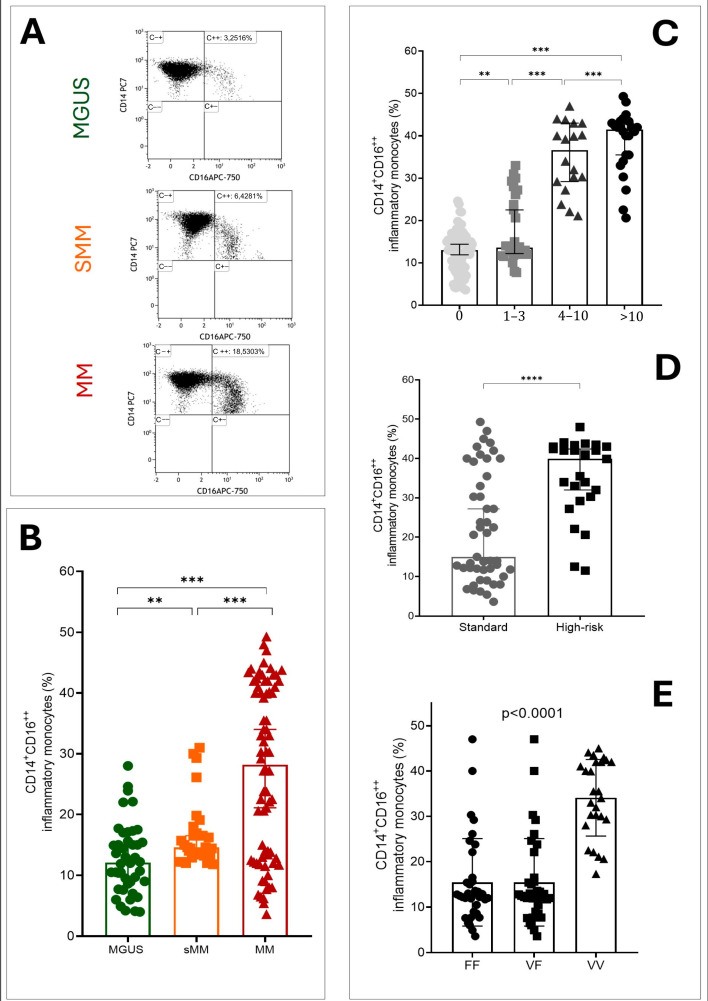
Inflammatory monocytes are increased in multiple myeloma and are associated with the FcγRIIIA genotype. The gating strategy to identify inflammatory monocytes by flow cytometry in MGUS, SMM and MM is shown in panel (**A**). (**B**) Panel shows the frequency of inflammatory monocytes in MGUS, SMM and MM. The frequency of inflammatory monocytes based on the number of lytic lesions detectable by WB-CT scan (**C**). The frequency of inflammatory monocytes based on cytogenetic risk in MM (we considered high-risk cytogenetic lesions: del 17p, t (4;14), t (14;16), t (4;14), amp 1q as detected by FISH)—(**D**). The frequency of inflammatory monocytes based on FcγRIIIA genotype (**E**). Bars represent median and interquartile ranges, ** *p* < 0.05, *** *p* < 0.01, **** *p* < 0.0001.

Given the difference in bone disease extension based on FcγR3A genotypic variants, we investigated if CD16a was differentially expressed on monocytes identifiable as circulating osteoclast precursors [23], by flow cytometry according to the gating strategy shown in Figure 4A. All subjects were devoid of immune-mediated diseases and acute or chronic viral infections.

A progressive increase in CD14^+^CD16^++^ non-conventional inflammatory monocytes (IM) was evident in MGUS through MM. IMs in MM patients were higher than in SMM or MGUS subjects (respectively, median and 5–95% interquartile range: 28.2% (IQR 6.4–45.7%) vs. 14.6% (IQR 11.9–30.4%) vs. 12.1% (IQR 4.1–24.5%), *p* < 0.0001) as shown in Figure 4B.

Among patients affected by symptomatic MM, IMs were significantly higher in patients with more than 10 lesions vs. 4–10 vs. 1–3 vs. no lesions (respectively: median and 5th–95th percentile range: 41.5% (IQR 21.1–48.9%) vs. 36.6% (IQR 21.1–47.0%) vs. 13.6% (IQR 7.8–32.1%) vs. 13% (4.2–22.1%), *p* < 0.0001), as shown in Figure 4C.

Among 72 patients with active MM who performed successfully FISH at diagnosis, the high-risk cytogenetics group (*N* = 25) carried higher percentage of IMs compared to the standard risk group (N = 47), respectively, 39.9% (IQR 11.8–46.8%) vs. 11.7% (IQR 5.7–46.2%), *p* < 0.0001, as shown in Figure 4D. In unadjusted analyses pooling MGUS, SMM and MM, V/V homozygous patients showed higher percentages of CD14^+^CD16^++^ cells than V/F or F/F (*p* < 0.001), as shown in Figure 4E. The genotype–monocyte comparison in Figure 4E was unadjusted and pooled across disease stages; residual confounding by disease status, bone disease burden, and cytogenetic risk cannot be excluded.

## 4. Discussion

In this work, we aimed to investigate whether polymorphisms in FcγR3A in plasma cell dyscrasias, or its expression in monocyte subsets, are associated with the extent of bone disease in MM.

For FcγR3A-V158F, a statistically significant association with the degree of bone disease emerged, with the FF genotype more frequent in patients with low extent of lesions, and the VV genotype more present in patients with advanced or aggressive disease. Indeed, patients carrying the FcγR3A F/F homozygous genotype had lower concentration of CD8 T-cells and resting memory CD4 T-cells, while carrying at least one V allele was significantly associated with lower percentage of M0 macrophages [24].

FcγR3A is mainly found on natural killer cells and macrophages, where it activates antibody-dependent cellular cytotoxicity to eliminate antibody-coated target cells [25]. FcγR3A-V158F polymorphism, resulting in an amino acid substitution of valine (V) to phenylalanine (F) at position 158 of the FcγR3a protein, leads to reduced affinity for IgG1 antibodies, but sialylation does not appear to impair binding to FcγRs [26,27].

MM is characterized by high levels of monoclonal IgG, with IgG1 as the most representative M-protein in MGUS and MM patients [28,29], while the formation of immune complexes depends on the integrity of the antibody [30]. If present, immune complexes can engage FcγRs on osteoclast precursors and macrophages, driving inflammatory cytokine production (IL-6, TNF-α) and amplifying bone destruction [31]. M-protein isolated from MM patients with bone disease has reduced IgG-Fc glycosylation and promotes osteoclast differentiation [7]. In MM, both α2,3- and α2,6-sialylation are decreased within complex-type N-glycans compared to controls [32], associated with immune-evasion and dysfunctional NK cells [33], with emerging therapeutic implications [34].

In osteolytic lesions of autoimmune disease, such as rheumatoid arthritis, the activating FcγRIII receptor sequesters the signal-transducing FcRγ subunit, preventing its association with other ITAM-containing receptors like PIR-A and OSCAR. This sequestration ultimately inhibits osteoclastogenesis by blocking the stimulatory signal that these other receptors would normally provide [35]. FcγRIIIA signaling enhances osteoclast differentiation via immune complex engagement, synergizes with RANKL and M-CSF pathways, and promotes osteoclastogenesis. Moreover, patients affected by rheumatoid arthritis with the 158V allele endure severe bone erosion compared with those with the 158F allele [36,37].

Our study does not provide clear evidence that a specific polymorphism of FcγR3A is associated with increased risk of MM progression but suggests that specific genotypes can contribute to accelerating phenomena like chronic inflammation and immune senescence onset. Indeed, patients carrying the FcγR3A V/V homozygous genotype had a significant increase in a circulating pro-inflammatory subset of monocytes.

CD14^+^CD16^+^ monocytes, often referred to as inflammatory monocytes, increase in number with age and contribute to immunosenescence [38,39]. They produce high levels of pro-inflammatory cytokines such as TNF-α and IL-6 [40], driving chronic inflammation (also known as inflammaging) [41] and are associated with age-related diseases like atherosclerosis [42] and extensive bone lesions in MM [43,44]. Despite their inflammatory profile, these monocytes may also have reduced functional capacity [41,45], making them both markers and mediators of age-related immune decline [46]. Recent reports suggest that CD14^+^CD16^+^ monocytes can contribute to immune dysfunction in MM due to their immunosuppressive activity on CD8^+^ T cells in the tumor microenvironment [47,48]. MM and its precursor states show immune aging signatures [49], given that immune age (inferred from immune cell profiles) correlates with disease severity, poorer vaccine responses, and differs by racial and ancestral backgrounds in MM and its precursors MGUS and SMM [49], where carrying a specific FcγR3A genotype could play a major role, independently of tumor load [50]. One possible scenario is that the FcγR3A genotype can affect the interaction between immune complexes and dendritic cells, which is widely considered to enhance antigen uptake, to internalize more protein antigens and cells [51], bacteria [52] when associated with IgG compared to free material.

The significant association observed in our cohort between the FcγRIIIA-158V/V genotype and RAS pathway mutations may have profound implications for disease dissemination. Extramedullary myeloma is characterized by near-universal alterations in the MAPK pathway, suggesting that these mutations are requisite for niche-independent clonal expansion [53]. RAS pathway mutations (KRAS, NRAS, BRAF) are the most frequent mutations in MM and are significantly enriched in patients with extramedullary spread and plasma cell leukemia [54]. RAS-mutated clones exhibit reduced dependency on the bone marrow niche through the constitutive activation of the MAPK pathway and the subsequent downregulation of adhesion molecules and chemokine receptors (e.g., CXCR4). This transition from a niche-dependent to a niche-independent state is a hallmark of extramedullary disease [55,56].

Taken together our findings suggest that the germline-encoded high-affinity FcγRIIIA-158V/V genotype may foster an inflammatory microenvironment that acts as a selective crucible for the emergence of more aggressive RAS/BRAF/MAPK-mutated clones, ultimately predisposing patients to a more invasive clinical phenotype.

The present study has some limitations that must be considered. Although our cohort is relatively large, subgroup analyses, such as less frequent genotype combinations, reduce the statistical power of the analysis. Furthermore, we did not directly assess the biological impact of the variants, such as by measuring IgG receptor affinity or the ability to mediate ADCC. Because FcγR3A is a germline locus, the observed cross-sectional associations across MGUS, SMM, and MM are biologically plausible; however, they do not demonstrate temporal causality or predict progression without longitudinal validation. Since our dataset is cross-sectional, longitudinal follow-up is required to test whether genotype and monocyte profiles independently predict time-to-progression. The association between FCGR3A VV and RAS/RAF carriage was derived from unadjusted comparisons and may be confounded by disease stage and tumor fraction; adjusted and stratified analyses are required to validate this signal.

In the future, it will be beneficial to expand the patient cohort to include independent controls, integrate functional analyses such as assessment of FcγR3A receptor expression or cytotoxicity assays, and adopt multiomic approaches that combine genomic, transcriptomic, and proteomic data.

## 5. Conclusions

Our study identifies the germline FcγRIIIA-158 V/V genotype as an emerging host-related biomarker of disease aggressiveness and genomic instability in plasma cell dyscrasias. The striking association between the high-affinity V/V variant and a higher mutational burden in the MAPK pathway (KRAS, NRAS, and BRAF) suggests how the host’s genetic immune background may actively shape the mutational landscape and clonal evolution of the tumor.

From a clinical perspective, the V/V genotype identifies a subset of patients characterized by a “high-velocity” phenotype, featuring advanced bone disease, high-risk cytogenetics, and severe anemia. If confirmed in larger prospective trials, incorporating FcγRIIIA genotyping into risk-stratification models could contribute to identifying “high-risk” MGUS and SMM patients who may benefit from earlier intervention or more intensive surveillance.

## Figures and Tables

**Table 1:** Patients’ characteristics based on disease status.

Characteristics	MGUS *N* = 42	SMM *N* = 31	MM *N* = 72	*p*-Value
Age				0.62
Years,	69.5	65.1	69.5	
median (IQR)	(38.4–83.0)	(50.5–83.0)	(52.1–84.4)	
AMC, cells × 10^3^/mmc,	0.5	0.47	0.39	0.06
Median (IQR)	(0.28–0.85)	(0.24–0.64)	(0.005–1.28)	
ALC, cells × 10^3^/mmc,	1.6	1.8	1.8	0.88
median (IQR)	(1–3.9)	(1.2–2.6)	(0.6–3.8)	
ANC cells × 10^3^/mmc,	4.2	3.8	4.1	0.77
median (IQR)	(1.9–9.6)	(2.6–8.3)	(1.7–9.3)	
Isotype, N (%)				0.89
IgD	0 (0)	0 (0)	1 (1.4)	
IgM	2 (4.8)	0 (0)	0 (0)	
IgG	30 (71.4)	26 (83.3)	40 (55.6)	
IgA	7 (16.7)	0 (0)	20 (27.8)	
λ	7 (16.7)	8 (25.8)	39 (54.2)	
K	31 (73.8)	19 (61.3)	28 (38.9)	
Not secretory	1 (2.4)	4 (12.9)	4 (5.6)	
FISH testing available, N (%) (when not available was due to low number of plasma cells)	26 (61.9)	20 (64.5)	60 (83.3)	NA
High-risk cytogenetics *, N (%)	0 (0)	2 (6.5)	25 (34.7)	<0.0001
Gender, N (%)				0.85
Male	20 (48)	21 (68)	43 (60)	
Female	22 (52)	10 (32)	29 (40)	
Hb (g/dL),	13.6	13.5	10.4	<0.0001
median (IQR)	(11.1–16.1)	(10.1–16.8)	(6.4–13.3)	
Serum albumin (g/dL),	3.9	3.8	3.6	0.62
median (IQR)	(3.1–4.2)	(3.5–4.6)	(2.5–4.3)	
β2-microglobulin (mg/L)	2.1	2.3	4.9	<0.0001
median (IQR)	(1.6–8.8)	(1.7–6.6)	(2.2–16.8)	
LDH, UI/L	177.5	192.0	208.5	0.67
median (IQR)	(129.1–281)	(130.0–277.5)	(129.7–325.2)	
BM clonal plasma cells (%)	5	15	60	<0.0001
median (IQR)	(3–10)	(10–40)	(25–90)	
M-protein concentration serum	0.25	1.17	2.12	<0.0001
median (IQR)	(0.1–2.8)	(0.1–3.9)	(0.5–8.1)	
Involved serum-free light chain concentration,	23.7	51.5	194.3	<0.0001
median (IQR)	(7.4–262.0)	(5.5–900.9)	(2.4–3900)	
Involved/uninvolved serum-free light chain concentration ratio	1.1	1.9	20.8	<0.0001
median (IQR)	(0.13–13.1)	(0.76–109.2)	(0.06–2480)	

* *17p13 deletion, 1q gain, t(4;14)*.

**Table 2:** Clinical variables in whole cohort based on FcγR3AV158F genotype.

Characteristics	FcγR3AV158F Genotype			
	F/F *N* = 35	V/F *N* = 84	V/V N = 26	*p*-Value
AMC, cells × 10^3^/mmc,	0.4	0.5	0.4	0.89
median (IQR)	(0.3–0.5)	(0.3–0.6)	(0.2–0.5)	
ANC cells × 10^3^/mmc,	4.2	4.0	4.1	0.98
median (IQR)	(3.3–5.9)	(3.2–5.5)	(3.3–5.6)	
ALC, cells × 10^3^/mmc,	1.6	1.8	1.5	0.86
median (IQR)	(1.3–3.9)	(1.3–2.2)	(1.2–2.4)	
Isotype, N (%)				0.23
IgG	25 (71.4)	53 (63.1)	13 (50.0)	
Non-IgG	10 (28.6)	31 (36.9)	13 (50.0)	
Hb (g/dL),	12.6	12.8	10.3	0.002
median (IQR)	(10.9–13.7)	(10.4–17.0)	(9.2–11.8)	
Serum albumin (g/dL),	3.6	3.8	3.6	0.89
median (IQR)	(3.1–4.1)	(2.6–4.5)	(2.7–4.4)	
β2-microglobulin (mg/L)	2.9	3.0	4.5	0.01
median (IQR)	(1.9–8.6)	(1.7–12.7)	(2.1–20.7)	
LDH, UI/L	192.0	194.5	224.5	0.02
median (IQR)	(159.1–313.2)	(144.3–227.8)	(167.5–263.5)	
Bone disease				<0.0001 * Chi-square test
0–3 lesions	30 (85.7)	69 (82.1)	4 (15.4)	
4 or more lesions	5 (14.3)	15 (17.9)	15 (84.6)	
Disease status				0.0001
MGUS	17 (40.5)	25 (54.8)	2 (4.8)	
SMM	4 (12.9)	25 (80.0)	2 (6.5)	
MM	14 (19.4)	36 (50.0)	22 (30.6)	
High-risk cytogenetics, *N* (%)	2 (5.7)	9 (10.7)	14 (53.8)	<0.0001
Mutational status CD138 cells,				<0.0001
*N*(%) *				
*NRAS, KRAS or BRAF* mutated	2 (20.0)	5 (22.7)	6 (66.6)	
*NRAS, KRAS and BRAF* wt	8 (80.0)	17 (77.3)	3 (33.4)	

* data available for 41 pts.

**Table 3:** Clinical variables in the cohort of 72 newly diagnosed MM patients based on FcγR3AV158F genotype.

Characteristics	FcγR3AV158F Genotype			
	F/F *N* = 14	V/F *N* = 36	V/V *N* = 22	*p*-Value
AMC, cells × 10^3^/mmc,	0.4	0.4	0.4	0.89
median (IQR)	(0.1–0.5)	(0.1–1.2)	(0.1–0.7)	
ANC cells × 10^3^/mmc,	5.4	3.9	4.1	0.98
median (IQR)	(1.3–13.4)	(2.3–6.9)	(2.1–7.1)	
ALC, cells × 10^3^/mmc,	1.7	2.0	1.5	0.86
median (IQR)	(0.6–3.4)	(0.9–3.1)	(0.7–3.1)	
Isotype, N (%)				0.23
IgG	10 (71.4)	20 (55.6)	10 (45.5)	
Non-IgG	4 (28.6)	16 (44.4)	12 (54.5)	
Hb (g/dL),	11.1	10.5	9.8	0.002
median (IQR)	(10.1–13.3)	(9.3–11.5)	(7.3–11.3)	
Serum albumin (g/dL),	3.6	3.6	3.5	0.89
median (IQR)	(3.1–3.7)	(2.8–4.2)	(3.1–4.3)	
β2-microglobulin (mg/L)	2.9	5.2	6.2	0.01
median (IQR)	(2.3–4.1)	(2.8–11.6)	(2.9–16.9)	
LDH, UI/L	194.0	204.5	230.5	0.02
median (IQR)	(136.1–299.0)	(132.0–254.0)	(153.4–398.1)	
Bone disease				0.02 *Chi-square test*
0–3 lesions	9 (64.3)	21 (58.3)	0 (0)	
4 or more lesions	5 (35.7)	15 (41.7)	22 (100)	
High-risk cytogenetics, *N* (%) *	2 (14.3)	9 (25.0)	14 (63.6)	<0.0001
Extramedullary disease, N (%)	2 (14.3)	5 (13.9)	6 (27.3)	0.04

* *17p13 deletion, 1q gain, t(4;14)*.

**Table 4:** Mutational panel analysis results.

		Disease Status				EMD (MM Only)		
	Total (N = 41)	MGUS-SMM (N = 21)	MM (N = 20)	*p* Value	Total (N = 20)	No (N = 14)	Yes (N = 6)	*p* Value
**NRAS**	6 (14.6%)	1 (4.8%)	5 (25.0%)	0.093	5 (25.0%)	3 (21.4%)	2 (33.3%)	0.613
**BRAF**	5 (12.2%)	1 (4.8%)	4 (20.0%)	0.184	4 (20.0%)	2 (14.3%)	2 (33.3%)	0.549
**KMT2D**	4 (9.8%)	3 (14.3%)	1 (5.0%)	0.606	1 (5.0%)	1 (7.1%)	0 (0.0%)	1.000
**DIS3**	3 (7.3%)	0 (0.0%)	3 (15.0%)	0.107	3 (15.0%)	2 (14.3%)	1 (16.7%)	1.000
**DUSP2**	3 (7.3%)	2 (9.5%)	1 (5.0%)	1.000	1 (5.0%)	1 (7.1%)	0 (0.0%)	1.000
**SETD2**	3 (7.3%)	0 (0.0%)	3 (15.0%)	0.107	3 (15.0%)	3 (21.4%)	0 (0.0%)	0.521
**FGFR3**	2 (4.9%)	1 (4.8%)	1 (5.0%)	1.000	1 (5.0%)	1 (7.1%)	0 (0.0%)	1.000
**KRAS**	2 (4.9%)	1 (4.8%)	1 (5.0%)	1.000	1 (5.0%)	1 (7.1%)	0 (0.0%)	1.000
**PRDM1**	2 (4.9%)	2 (9.5%)	0 (0.0%)	0.488	0 (0.0%)	0 (0.0%)	0 (0.0%)	1.000
**SP140**	2 (4.9%)	1 (4.8%)	1 (5.0%)	1.000	1 (5.0%)	1 (7.1%)	0 (0.0%)	1.000
**ZNF292**	2 (4.9%)	1 (4.8%)	1 (5.0%)	1.000	1 (5.0%)	1 (7.1%)	0 (0.0%)	1.000
**BHLHE41**	1 (2.4%)	1 (4.8%)	0 (0.0%)	1.000	0 (0.0%)	0 (0.0%)	0 (0.0%)	1.000
**CYLD**	1 (2.4%)	0 (0.0%)	1 (5.0%)	0.488	1 (5.0%)	1 (7.1%)	0 (0.0%)	1.000
**H1-4**	1 (2.4%)	0 (0.0%)	1 (5.0%)	0.488	1 (5.0%)	0 (0.0%)	1 (16.7%)	0.300
**IGLL5**	1 (2.4%)	0 (0.0%)	1 (5.0%)	0.488	1 (5.0%)	1 (7.1%)	0 (0.0%)	1.000
**KLHL6**	1 (2.4%)	0 (0.0%)	1 (5.0%)	0.488	1 (5.0%)	0 (0.0%)	1 (16.7%)	0.300
**PRKD2**	1 (2.4%)	1 (4.8%)	0 (0.0%)	1.000	0 (0.0%)	0 (0.0%)	0 (0.0%)	1.000
**RASA2**	1 (2.4%)	0 (0.0%)	1 (5.0%)	0.488	1 (5.0%)	1 (7.1%)	0 (0.0%)	1.000
**RPL5**	1 (2.4%)	1 (4.8%)	0 (0.0%)	1.000	0 (0.0%)	0 (0.0%)	0 (0.0%)	1.000
**TCL1A**	1 (2.4%)	1 (4.8%)	0 (0.0%)	1.000	0 (0.0%)	0 (0.0%)	0 (0.0%)	1.000
**TENT5C**	1 (2.4%)	0 (0.0%)	1 (5.0%)	0.488	1 (5.0%)	1 (7.1%)	0 (0.0%)	1.000
**TRAF2**	1 (2.4%)	0 (0.0%)	1 (5.0%)	0.488	1 (5.0%)	0 (0.0%)	1 (16.7%)	0.300
**ZNF462**	1 (2.4%)	1 (4.8%)	0 (0.0%)	1.000	0 (0.0%)	0 (0.0%)	0 (0.0%)	1.000

## Data Availability

The data that support the findings of this study are available from the corresponding authors upon reasonable request.

## Abbreviations

The following abbreviations are used in this manuscript:

MM	Multiple myeloma
PC	Plasma cell
BM	Bone marrow
MGUS	Monoclonal gammopathy of uncertain significance
SMM	Smoldering myeloma
CT	Computed tomography
MRI	Magnetic resonance imaging
ICs	Immune complexes
ADCC	Antibody-dependent cellular cytotoxicity
PB	Peripheral blood
SDS	Sodium dodecyl sulfate
IMs	Non-conventional inflammatory monocytes
